# Impacts of External Influences on Treatment in Emergency Psychiatry: Results of a Longitudinal Measurement in an Adult Psychiatry Clinic in Germany

**DOI:** 10.1192/j.eurpsy.2023.647

**Published:** 2023-07-19

**Authors:** T. Barth, S. Voigtlaender

**Affiliations:** Psychiatry and psychotherapy, Klinikum Chemnitz, Chemnitz, Germany

## Abstract

**Introduction:**

Regarding last years, in German psychiatry the effects of the changed statutory framework conditions for the use of physical restraints [1,2,3] and the COVID-19 pandemic on the treatment in emergency psychiatry were discussed.

**Objectives:**

Against these background, changes in the severity of disease and regarding the use of coercive measures in our emergency psychiatry are to be analysed.

**Methods:**

An internal retrospective study in the emergency psychiatry (2017=reference period; 2019=post changed statutory framework; 2021=post changed statutory framework and during the pandemic) was performed.

**Results:**

- The socio-demographic patient data (exception: gender) and the distribution of the main diagnoses groups remained stable. There was a reduction in the treatment volume by 4% in the pandemic period compared to 2019.

- Both in 2019 and 2021, significant increases regarding the number of patient characteristics of the intensive treatment according to the OPS code 9-61 [7,8,9] were measured.

- During pandemic period 2021, a significantly rise in the percentage of involuntarily committed treatment cases [10] imposed.

- Following changed framework conditions, there were decreases in the total duration of physical restraint and the number of restraint events per restrained treatment case [10] ratio of five-point/ seven-point restraint events reduced significantly and continuously.

**Conclusions:**

The amendments in statutory framework for the use of physical restraints made personnel more aware of the issue and consequently led to changes in restraint practice at our emergency psychiatric unit. These effects were partially cancelled by the increases in the severity of diseases during the pandemic.

**Disclosure of Interest:**

None Declared

